# Assessing Multivariate Constraints to Evolution across Ten Long-Term Avian Studies

**DOI:** 10.1371/journal.pone.0090444

**Published:** 2014-03-07

**Authors:** Celine Teplitsky, Maja Tarka, Anders P. Møller, Shinichi Nakagawa, Javier Balbontín, Terry A. Burke, Claire Doutrelant, Arnaud Gregoire, Bengt Hansson, Dennis Hasselquist, Lars Gustafsson, Florentino de Lope, Alfonso Marzal, James A. Mills, Nathaniel T. Wheelwright, John W. Yarrall, Anne Charmantier

**Affiliations:** 1 Département Ecologie et Gestion de la Biodiversité UMR 7204 CNRS/MNHN/UPMC, Muséum National d'Histoire Naturelle, Paris, France; 2 Department of Biology, Lund University, Ecology Building, Lund, Sweden; 3 Laboratoire d'Ecologie, Systématique et Evolution, CNRS UMR 8079, Université Paris-Sud, Orsay, France; 4 Department of Zoology, University of Otago, Dunedin, New Zealand; 5 Department of Zoology, Biology Building, University of Seville, Seville, Spain; 6 Department of Animal and Plant Sciences, University of Sheffield, Sheffield, United Kingdom; 7 Centre d'Ecologie Fonctionnelle et Evolutive UMR 5175 CNRS, Montpellier, France; 8 Department of Animal Ecology, Evolutionary Biology Center, Uppsala University, Uppsala, Sweden; 9 Departamento de Zoología, Universidad de Extremadura, Badajoz, Spain; 10 Corning, New York, United States of America; 11 Department of Biology, Bowdoin College, Brunswick, Maine, United States of America; 12 Lincoln, Christchurch, New Zealand; University of Idaho, United States of America

## Abstract

**Background:**

In a rapidly changing world, it is of fundamental importance to understand processes constraining or facilitating adaptation through microevolution. As different traits of an organism covary, genetic correlations are expected to affect evolutionary trajectories. However, only limited empirical data are available.

**Methodology/Principal Findings:**

We investigate the extent to which multivariate constraints affect the rate of adaptation, focusing on four morphological traits often shown to harbour large amounts of genetic variance and considered to be subject to limited evolutionary constraints. Our data set includes unique long-term data for seven bird species and a total of 10 populations. We estimate population-specific matrices of genetic correlations and multivariate selection coefficients to predict evolutionary responses to selection. Using Bayesian methods that facilitate the propagation of errors in estimates, we compare (1) the rate of adaptation based on predicted response to selection when including genetic correlations with predictions from models where these genetic correlations were set to zero and (2) the multivariate evolvability in the direction of current selection to the average evolvability in random directions of the phenotypic space. We show that genetic correlations on average decrease the predicted rate of adaptation by 28%. Multivariate evolvability in the direction of current selection was systematically lower than average evolvability in random directions of space. These significant reductions in the rate of adaptation and reduced evolvability were due to a general nonalignment of selection and genetic variance, notably orthogonality of directional selection with the size axis along which most (60%) of the genetic variance is found.

**Conclusions:**

These results suggest that genetic correlations can impose significant constraints on the evolution of avian morphology in wild populations. This could have important impacts on evolutionary dynamics and hence population persistence in the face of rapid environmental change.

## Introduction

With the realisation that evolution can occur rapidly, there has been growing interest in measuring short term microevolutionary responses to natural selection and attempting to predict such responses in wild populations [1]. Understanding responses to selection is an exciting challenge, both at the fundamental level when attempting to understand and predict evolutionary mechanisms and at the applied level, as in the case of management of responses to anthropogenic changes such as global warming [2]. However, the relevance of our predictions for evolutionary trajectories in natural settings will depend on how accurately we can assess selective pressures and evolutionary potential.

Evolutionary potential is often estimated as heritability (h^2^). However, most studies have reported a discrepancy between predicted and observed evolutionary responses to selection when using the breeder's equation [3], [4], where heritability is multiplied by selection to obtain the expected evolutionary response [5]. One of the possible explanations for this discrepancy is that the estimates of evolutionary potential are inaccurate [6]. A major limitation of equating the evolutionary potential of a character with its heritability comes from the fact that phenotypes result from the interaction of several characters that are functionally, developmentally and genetically linked. Approaching phenotypes as a set of independent traits may thus give a very misleading picture of expected phenotypic responses to selection [7], [8]. Hence, estimating evolutionary potential requires understanding and assessing how genetic architecture, notably genetic correlations between traits, influences responses to selection, either by constraining or by facilitating such responses [9], [10].

**Figure 1:**
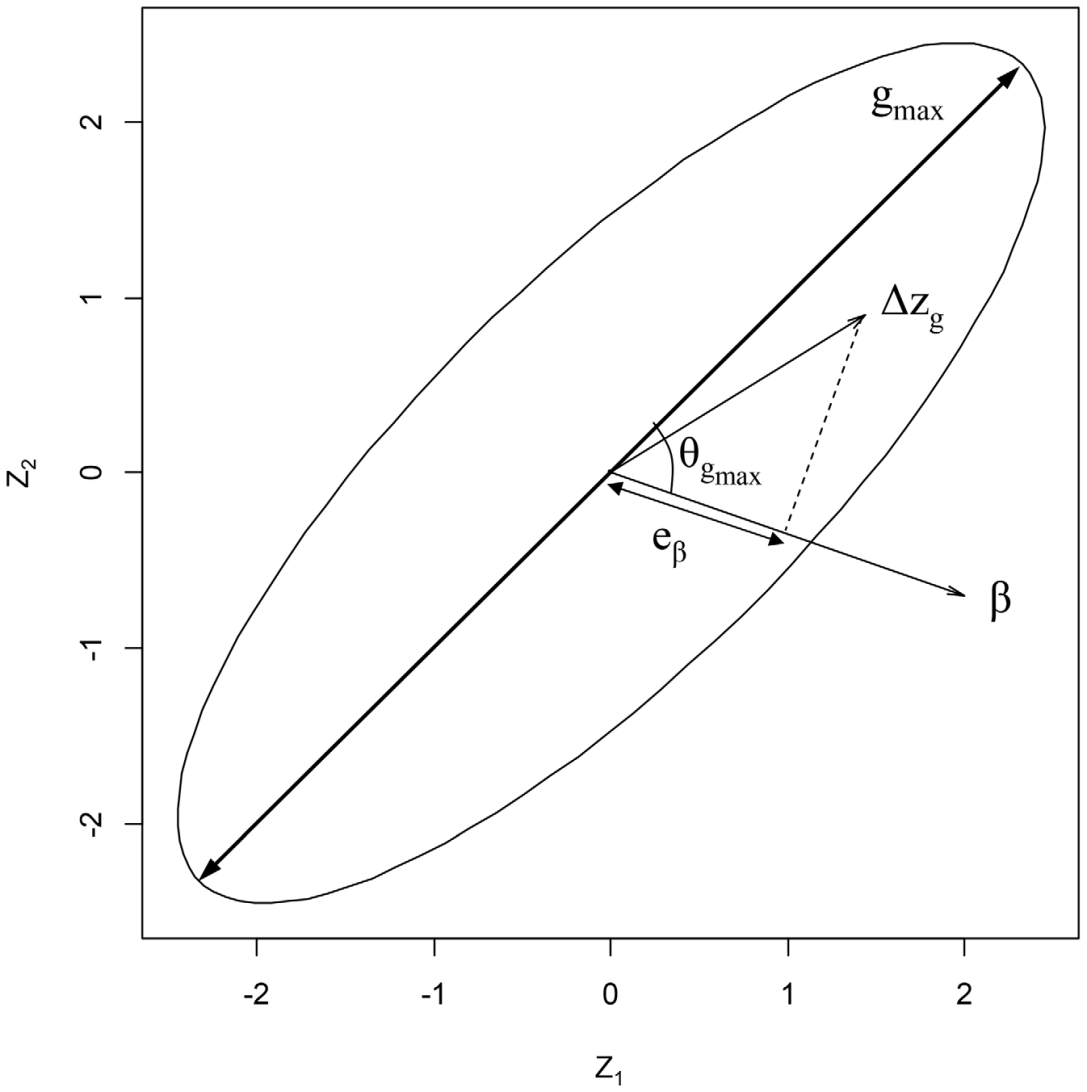
Measures of constraints on response to selection for two traits, z_1_ and z_2_. G is represented by the ellipse, g_max_ is the first eigenvector of G, β is the vector of directional selection, and Δz_g_ is the response to selection calculated from the multivariate breeder's equation in the presence of genetic correlations. e_β_, the multivariate evolvability, is the projection of the response to selection on β. θ_gmax_ is the angle between g_max_ and β. Redrawn from [12].

Technically, such an assessment implies the estimation of genetic correlations as well as selection on correlated characters. The **G** matrix, the matrix of additive genetic variances and covariances, summarizes the genetic architecture for a set of traits. A geometrical representation of the **G** matrices can help to visualize how the information contained in the **G** matrices can be interpreted in terms of evolutionary potential for a given set of traits in a population. A spherical **G** matrix (with equal amount of additive genetic variance in all directions) provides an opportunity for the same amount of evolutionary response in all directions of phenotypic space. An elliptical **G** matrix, on the other hand, is characterized by a main axis of additive genetic variance (**g_max_**, Fig. 1, [11]). This axis represents the direction of highest evolvability in the phenotypic space, hence the direction in which an evolutionary response is facilitated [12]. If selection and **g_max_** are not aligned, the response to selection will be slower and evolvability reduced. Multivariate evolutionary constraints arise when there is little or no genetic variation in the direction of selection i.e. if some traits are genetically negatively correlated but submitted to similar (positive or negative) selection pressures, or if traits are positively correlated but submitted to antagonistic selection pressures.

Recently, Agrawal & Stinchcombe [13] reviewed the impact of genetic correlations on the predicted rate of adaptation, gathering results from 45 studies on various plant and animal species, but found no general pattern: genetic correlations could either constrain or facilitate response to selection. However, two recent population-based studies that used the rate of adaptation metric defined by Agrawal & Stinchcombe ([13], see Methods) found that genetic covariances could decrease the rate of adaptation of life history traits by as much as 50% [14], [15]. Hence, although it is difficult to generalize the impact of **G** on the response to selection, recent studies show that genetic constraints to evolution can be very strong. Presently, the scope for comparison of such metrics of multivariate constraints is very limited. As a result, more empirical work is needed across a range of species and traits in order to reach general conclusions about the influence of the **G** matrix on adaptation [16].

This study aims to generalize our knowledge of constraints or facilitation on the evolution of morphological traits, and to investigate how the interplay between selection and **G** matrices leads to such constraints. Our main objective is to estimate the impact of genetic correlations on evolutionary trajectories using a comparative approach based on data from long-term field studies (>12 years) of 10 populations of seven bird species. We chose four morphological traits (body mass, and length of tarsus, bill and wing, *i.e*., traits that represent shape and body size), because we were interested in assessing potential constraints for traits known to harbour considerable genetic variation e.g. [5], [17], [18]. First, we evaluate how **G** affects the predicted relative rate of adaptation, R_A_ [13] for these morphological characters. The rate of adaptation is the expected fitness gain due to an evolutionary response to current selection. The relative rate of adaptation (R_A_) is obtained by comparing the rate of adaptation under models using observed **G** versus models with genetic correlations fixed to zero. Second, we seek to understand the origin of such patterns (1) by comparing the multivariate evolvability in the direction of the estimated directional selection (e_β_, which corresponds to the amount of predicted evolutionary response in the exact direction of current directional selection, **β**) versus the average evolvability in random directions of the phenotypic space, 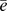 [12], [19]; and (2) by determining the orientation of the axis containing the highest percentage of additive genetic variance relative to the direction of selection (Fig. 1, [20]). This can be done by assessing the angle (θ_gmax_) between **g_max_** and the directional selection **β**. These evaluations will allow the assessment of the extent to which a predicted micro-evolutionary response is facilitated or constrained by genetic correlations.

## Methods

### Ethical statement

All data came from authorized monitoring of natural populations and did not involve keeping birds in captivity. Such long-term studies require that birds are subject to minimal disturbance, and no manipulation was performed that would have caused animal suffering. Furthermore, all studies complied with national and international guidelines. All people collecting the data had banding permits.

### Species and focal traits

Investigating evolutionary processes resulting from natural selection requires the use of data sets where phenotypes and relatedness are collected from populations in their natural environment. In this situation, estimating accurate **G** matrices necessitates long-term datasets with multigenerational pedigrees. We focused on four morphological traits that are most commonly measured in adult birds: wing length, tarsus length, body mass and bill length. Populations were hence selected based on the availability of a pedigree and the minimum number of morphological traits needed. We gathered 10 data sets representing seven bird species from three continents (Table 1): red-billed gull (*Chroicocephalus scopulinus*, [21]), great reed warbler (*Acrocephalus arundinaceus*, [22]), barn swallow (*Hirundo rustica*, two populations, [23]), blue tit (*Cyanistes caeruleus*, three populations, [24]), collared flycatcher (*Ficedula albicollis*, [25]), Savannah sparrow (*Passerculus sandwichensis*, [26]) and house sparrow (*Passer domesticus*, [27]).

**Table 1:** Summary of basic information for each population: Percentage of individuals of unknown age in the sample, size of the pedigrees and amount of data available for each population.

				% of individuals of unknown age	Pruned pedigree		Number of observations (Number of individuals)			
Family	Species		Population		Number of Individuals	Number of Generations	Wing length	Tarsus length	Mass	Bill length
Laridae	Red billed gull		1- Kaikoura, New Zealand	31	4965	5	3080 (2442)	1628 (1415)	5682 (4530)	6173 (4858)
Sylviidae	Great reed warbler		2- Kvismaren, Sweden	0	551	7	918 (540)	808 (492)	869 (515)	646 (413)
Hirundinidae	Barn swallow		3- Badajoz, Spain	0	1407	4	2318 (1399)	2272 (1375)	2290 (1384)	2278 (1375)
			4- Kraghede, Denmark	1	487	3	561 (487)	560 (487)	561 (487)	521 (447)
Paridae	Blue tit		5- Muro, France	34	921	6	1303 (899)	1312 (899)	1304 (894)	1254 (866)
			6- Pirio, France	30	1124	11	2016 (1079)	1705 (937)	2081 (1104)	1647 (924)
			7- Rouviere, France	13	1056	9	1797 (1045)	1786 (1042)	1803 (1043)	1744 (1022)
Muscicapidae	Collared flycatcher		8- Gotland, Sweden	0.08	6731	14	9341 (6299)	9183 (6218)	9286 (6253)	6133 (4409)
Emberizidae	Savannah sparrow		9- Kent Island, Canada	0	1538	11	1965 (1487)	1686 (1302)	1791 (1361)	1360 (1079)
Passeridae	House sparrow		10- Lundy, UK	11	391	9	780 (360)	778 (360)	766 (358)	630 (292)

Wing length is a trait connected to flight performance and is especially important in migratory species [28] such as those included in this study (collared flycatcher, barn swallow, great reed warbler, Savannah sparrow). Tarsus length is a good approximation for overall structural size in birds, because it is a skeletal measurement [29]. Body mass is also a general size measure, but more condition-dependent than tarsus length. Balbontín et al. [30] showed that body mass reflects condition, and that as such it provides a measure of changing condition among age classes and generations. Bill length is associated with many characters, including foraging and song performance [31], [32]. All of these traits have been shown to be heritable in several bird species [33]–[37].

### Estimation of the additive genetic (co)variance matrix

We estimated the **G** matrix in each population by using multivariate animal models [38], [39]. Random effects included additive genetic effects (V_A_) and permanent environmental effects to account for repeated measurements of the same individual (V_PE_) as well as a year effect (V_year_). The analyses excluded measurements on offspring of the year. Age was included as a continuous variable (linear + quadratic) to account for aging effects on trait size. Tarsus length can change because of swelling or reduction of cartilage, wing feathers re-grow annually and are affected by aging and wear, beak length may become worn depending on diet, and body mass can be affected by age, e.g., because of decreased feeding performance. As we wanted to avoid losing power by removing individuals of unknown age (portion given in Table 1), we used mean substitution for individuals of unknown age: age was mean-centred and those individuals were assigned an age of zero. Because of power issues and technical complexity, males and females were not analysed separately so models contained sex as a fixed effect. When available and significant, we included a polynomial date effect (degree 2 or 3, according to significance) to control for mass and bill length variation during the breeding season. This affected the residual (co) variances, but not estimates of **G**. To avoid traits with larger means (Table 2) exerting a disproportionate effect on general patterns, we standardised traits prior to analysis. Because scaling to phenotypic variance (which can vary independently of additive genetic variance) can lead to problems of interpretation [40], we used standardization to the trait's overall mean [12], [40].

**Table 2:** Summary of basic information for each population: Means and standard deviations for each trait.

		Wing length		Tarsus length		Mass		Bill length	
Species	Population	mean	std dev	mean	std dev	mean	std dev	mean	std dev
Red billed gull	1- Kaikoura, New Zealand	278.13	8.75	443.12	18.95	288.99	26.81	49.83	2.3
Great reed warbler	2- Kvismaren, Sweden	98.33	2.89	33.09	1.03	33.37	2.43	12.51	0.57
Barn swallow	3- Badajoz, Spain	120.52	3.08	11.15	0.53	18.33	1.56	7.53	0.55
	4- Kraghede, Denmark	125.68	2.97	11.18	0.81	19.28	1.52	7.82	0.49
Blue tit	5- Muro, France	61.83	2.02	16.24	0.51	9.71	0.48	9.76	0.48
	6- Pirio, France	62.1	2.05	16.04	0.51	9.31	0.5	9.74	0.45
	7- Rouviere, France	65.92	2.27	16.7	0.52	11.07	0.65	9.86	0.46
Collared flycatcher	8- Gotland, Sweden	81.76	2.19	19.37	0.59	13.68	1.33	13.36	0.82
Savannah sparrow	9- Kent Island, Canada	66.59	2.68	21.07	0.7	19.71	1.62	8.09	0.38
House sparrow	10- Lundy, UK	77.71	2.17	18.55	0.83	27.61	1.86	13.29	0.58

Measurements are in millimetres (mm) and mass in grams (g).

A simple description of the multivariate animal model for one population is as follows: 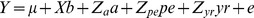
(1)where **Y** is the vector of standardised phenotypic observations for all individuals, **μ** is a vector of mean phenotypes, **b** is the vector of fixed effects to be fitted (age, sex and date), and **X** is the design matrix relating phenotypic observations to the vector of fixed effects. Fixed effects were individually chosen for each population based on significance levels in a preliminary analysis (Table S1). For the random effects, **a** is the vector of additive genetic values, **pe** the vector of permanent environment effects, and **yr** the vector of year of measurement effect, with Z_a_, Z_pe_ and Z_yr_ their respective design matrices. All random effects are assumed to be normally distributed, and elements of **a** are assumed to be drawn from 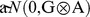 where **G** is the additive genetic variance-covariance matrix and **A** the relatedness matrix derived from the pedigree.

All pedigrees were pruned using the R package “pedantics” [41] so they contained only informative individuals [41]. Details for each population are given in Table 1 and Fig. S1.

### Estimating selection

To assess selection coefficients in each population, we used the classic approach by Lande & Arnold [42]. Directional selection gradients (**β**) were estimated by regressing relative fitness against morphological traits. Similarly, non-linear selection (**γ** matrix) gradients were estimated using quadratic regressions, including cross products between traits, representing correlational selection gradients. Quadratic coefficients from the regression were doubled so that they became analogous to selection coefficients [43].

Annual contribution to total individual fitness was estimated by yearly reproductive success (the number of fledged offspring). Morphological traits were first standardised by their means and then corrected for the same significant fixed effects as used in the animal models (i.e., effects of the fixed factors were subtracted from the actual measurement values), prior to selection analysis to obtain selection estimates consistent with the **G** matrices [12].

Each variable (fitness and morphological traits) was standardized within year, i.e. fitness was divided by annual population average success and we subtracted the mean annual phenotypic value from the overall mean standardized morphological variables.

### Estimating Constraints on and Facilitations of Responses to Current Selection

We estimated how genetic correlations could affect both evolutionary trajectories and relative rate of adaptation in each population. First, we estimated the impact of genetic correlations on the predicted rate of adaptation in order to assess constraints on or facilitation of a response to the current selection acting in the populations, using the metric R_A_ defined by Agrawal & Stinchcombe [13]. This metric is the ratio between the predicted change in fitness given the predicted evolutionary change in mean phenotype per generation, in the presence of genetic correlations relative to what it would have been without these correlations. It is defined as: 
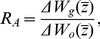
(2)with 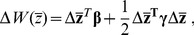
(3)where 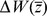 is the rate of adaptation (predicted change in fitness based on the predicted change of the mean phenotype of the population), 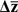 the predicted change in average phenotype in the population calculated using the breeder's equation 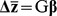, **β** the vector of directional selection gradients, and **γ** the matrix of non-linear selection. In equation (2), 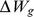 is the rate of adaptation taking into account genetic correlations, while 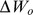 is the rate of adaptation when all the covariances between traits are set to 0. The ratio R_A_ is then compared to 1, with a ratio larger than 1 implying higher rate of adaptation in the presence of genetic correlations (facilitation), while a ratio lower than 1 implies that genetic correlations slow down adaptation (constraint, [13]). To estimate the overall means across populations (equivalent to a meta-analytic mean) for relative rate of adaptation R_A_, a ratio, we used the geometric mean: the overall mean was estimated for each iteration when estimating R_A_ for each population, so that a confidence interval could be built.

### Evolvabilities

We also estimated multivariate evolvability and average evolvability [12]. Multivariate evolvability is the amount of predicted evolutionary response occurring in the exact direction of selection (e_β_, Fig. 1). It is estimated as 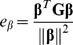
(4)


Average evolvability over random selection gradients [12] represents the evolutionary potential associated with the **G** matrix if averaged across all possible directions in the phenotypic space. It is defined as 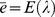
(5)where λs are the eigenvalues of **G**. Average evolvability thus does not depend on genetic correlations [12]. Note that our definitions of multivariate evolvabilities follow Hansen and Houle [12]. Evolvability can be defined as a univariate (variance scaled to the mean) or a multivariate estimate. Following [12], [40], we use “I_A_- evolvability” for univariate estimates of additive genetic variance scaled to the mean and “e” for multivariate estimates of evolvability.

### Angle between directional selection and g_max_

**g_max_** is the first eigenvector of **G** and the amount of additive genetic variance it contains is the eigenvalue of this vector. The sum of all eigenvalues of **G** represents the total additive genetic variance. Hence, the proportion of genetic variance along **g_max_** was estimated for each population by the ratio between the first eigenvalue of **G** and the sum of the four eigenvalues. This gives an assessment of the evenness of the distribution of the genetic variance in the different dimensions of **G**.

The angle between **g_max_** and the direction of selection (**β**, Fig. 1) estimates how close selection is from the axis that is the direction of least resistance. If selection and g_max_ are aligned, the response to selection will be maximal while it will be constrained with increasing angles (with maximum constrain at 90°). The angle between g_max_ and β was calculated using: 
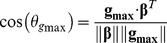
(6)


The angle between **g_max_** and **β** cannot exceed 90° because **g_max_** can be considered in its two opposite directions. Hence, if an angle larger than 90° was found, we took the complementary value 180-θ_gmax_.

### Estimation method

Both animal models and selection analyses were run using Bayesian methods with the MCMCglmm R Package [44]. The advantage of the Bayesian approach is that the use of posterior distributions facilitates the propagation of errors in estimates [45]. Although uncertainty around estimates of **G** matrices is usually large [8], attempts to integrate this uncertainty in the next steps (e.g., predicted response to selection) are extremely rare [14]. One of the goals of this analysis is to provide such estimates for each quantity described above.

The posterior distribution was a sample of 1000 values for each parameter. We used a total of 1,200,000 iterations for each analysis, with a burn-in phase of 200,000 and thinning of 1000. Priors were defined for variances and covariances. We assessed two priors for variances and covariances for each analysis: (1) a parameter expanded prior [46] and (2) a slightly informative prior (V  =  diag(n)*Vp/r, nu  =  n), where Vp is the phenotypic variance, n the number of traits and r the number of random factors. Our results were not sensitive to the choice of prior (Fig. S2).

In the main text, we chose to present results from the model with slightly informative priors as it has a direct biological interpretation: the prior specification implies that (1) the variance is distributed evenly across the random terms and (2) traits are independent [44]. If information is coming from the prior, as it is built with null covariances, estimated genetic covariances would be biased downwards, if anything, and our estimates conservative.

## Results

### G-matrices

I_A_-evolvabilities (100× additive genetic variance of traits scaled to the square of their mean), interpreted as the expected percentage of trait change per generation if it were submitted to selection as strong as on fitness itself [47], were on average 0.061% (range 0.013%–0.178%, Tables 3, 4 and 5) across traits and populations. Heritability estimates were on average 0.30 (range 0.05–0.60, Table S2). High I_A_-evolvabilities did not correspond to high heritabilities, and overall both estimates were unrelated (R^2^ = 0.04). The absence of congruence between evolutionary potential predicted from heritabilities and I_A_-evolvabilities is in line with a recent review [40].

**Table 3:** Estimates of mean standardized traits I_A_-evolvabilities (estimated V_A_×100) and genetic covariances (×100) for Red-billed gull, Great reed warbler, and the two Barn swallow populations with their 95% confidence interval.

	Red-billed gull			Great reed warbler			Barn swallow - Badajoz			Barn swallow - Kraghede		
	posterior mode	Low 95%CI	Up 95%CI	posterior mode	Low 95%CI	Up 95%CI	posterior mode	Low 95%CI	Up 95%CI	posterior mode	Low 95%CI	Up 95%CI
Wing	0.032	0.027	0.036	0.019	0.015	0.026	0.02	0.012	0.03	0.03	0.018	0.042
Tarsus	0.045	0.037	0.061	0.036	0.025	0.047	0.031	0.018	0.044	0.086	0.038	0.127
Mass	0.109	0.09	0.14	0.077	0.047	0.122	0.132	0.091	0.198	0.178	0.085	0.307
Bill	0.036	0.032	0.04	0.083	0.059	0.115	0.073	0.037	0.108	0.047	0.025	0.1
Wing:Tarsus	0.015	0.009	0.021	0.001	−0.003	0.009	0.004	−0.006	0.01	0.025	0.006	0.041
Wing:Mass	0.021	0.014	0.031	0.012	0.001	0.023	0.024	0.005	0.039	0.038	0.006	0.057
Wing:Bill	0.01	0.006	0.013	0.004	−0.006	0.013	0.004	−0.009	0.017	0	−0.015	0.024
Tarsus:Mass	0.038	0.025	0.052	0.027	0.01	0.042	0.022	0.001	0.042	0.031	−0.014	0.087
Tarsus:Bill	0.015	0.009	0.021	0.013	0.003	0.029	0.007	−0.009	0.024	0.006	−0.025	0.037
Mass:Bill	0.022	0.015	0.03	0.023	0.002	0.05	0.03	−0.003	0.064	0.007	−0.037	0.06

I_A_-evolvabilities were higher for mass than for other characters because of the cubic scale of this measurement, while other characters were measured on a linear scale [40].

**Table 4:** Estimates of mean standardized traits I_A_-evolvabilities (estimated V_A_×100) and genetic covariances (×100) for the three Blue tit populations with their 95% confidence interval.

	Blue tit - Muro			Blue tit - Pirio			Blue tit - Rouvière		
	posterior mode	Lower 95%CI	Upper 95%CI	posterior mode	Lower 95%CI	Upper 95%CI	posterior mode	Lower 95%CI	Upper 95%CI
Wing	0.018	0.013	0.026	0.013	0.009	0.018	0.02	0.015	0.024
Tarsus	0.041	0.027	0.054	0.043	0.029	0.058	0.043	0.035	0.054
Mass	0.075	0.042	0.11	0.079	0.053	0.11	0.103	0.077	0.131
Bill	0.045	0.028	0.068	0.028	0.017	0.041	0.05	0.038	0.065
Wing:Tarsus	0.01	0.003	0.017	0.012	0.005	0.017	0.007	0.002	0.011
Wing:Mass	0.012	0.002	0.025	0.012	0.005	0.022	0.017	0.009	0.025
Wing:Bill	0.008	−0.002	0.015	0.007	0	0.011	0.006	0	0.011
Tarsus:Mass	0.021	0.006	0.039	0.029	0.012	0.046	0.02	0.012	0.035
Tarsus:Bill	0.019	0.006	0.031	0.013	0.001	0.022	0.01	0.002	0.018
Mass:Bill	0.032	0.008	0.048	0.017	0.002	0.03	0.029	0.015	0.042

I_A_-evolvabilities were higher for mass than for other characters because of the cubic scale of this measurement, while other characters were measured on a linear scale [40].

**Table 5:** Estimates of mean standardized traits I_A_-evolvabilities (estimated V_A_×100) and genetic covariances (×100) for the Collared flycatcher, Savannah sparrow and House sparrow populations with their 95% confidence interval.

	Collared flycatcher			Savannah sparrow			House sparrow		
	posterior mode	Lower 95%CI	Upper 95%CI	posterior mode	Lower 95%CI	Upper 95%CI	posterior mode	Lower 95%CI	Upper 95%CI
Wing	0.018	0.015	0.02	0.02	0.015	0.027	0.024	0.017	0.032
Tarsus	0.037	0.033	0.043	0.036	0.025	0.047	0.09	0.051	0.135
Mass	0.115	0.095	0.133	0.117	0.081	0.177	0.174	0.108	0.241
Bill	0.045	0.039	0.056	0.087	0.058	0.109	0.09	0.06	0.13
Wing:Tarsus	0.007	0.005	0.01	0.005	0	0.013	0.027	0.014	0.046
Wing:Mass	0.015	0.009	0.019	0.015	0.005	0.032	0.036	0.023	0.061
Wing:Bill	0.006	0.003	0.01	0.01	−0.001	0.018	0.015	0.002	0.026
Tarsus:Mass	0.028	0.022	0.036	0.02	0.007	0.046	0.086	0.044	0.136
Tarsus:Bill	0.008	0.004	0.014	0.016	0	0.026	0.041	0.005	0.069
Mass:Bill	0.015	0.005	0.024	0.036	0.012	0.064	0.053	0.019	0.091

I_A_-evolvabilities were higher for mass than for other characters because of the cubic scale of this measurement, while other characters were measured on a linear scale [40].

Genetic covariances between all traits were positive in all populations (Tables 3, 4 and 5), and average genetic correlations were 0.35 (range: 0 to 0.76). In all populations, **g_max_** contained more than half of the total amount of additive genetic variance (geometric mean (95% CI): 61.3% (58, 64), Table 6) which suggests that **G** matrices were classically elliptical rather than spherical. The first eigenvalue, which represents maximal evolvability if selection and **g_max_** are aligned [12], was of the order of 0.1 to 0.2 (values ×100, Table 6). All traits loaded positively on **g_max_** (Table 6), and body mass consistently had the highest loading on **g_max_**. Because the first axis of a PCA can be interpreted as a size index, this suggests that the line of genetic least resistance (**g_max_**) is associated with body size.

**Table 6:** Percentage of variance along g_max_ and value of the first eigenvalue (×100) with 95% confidence intervals, and loading of the four morphological traits on g_max_.

	Percentage of variance along g_max_	First eigenvalue	Wing	Tarsus	Mass	Bill
Red billed gull	63.4 (58.28, 69.46)	0.143 (0.118, 0.173)	0.24	0.415	0.839	0.257
Great reed warbler	53.66 (44.69, 64.6)	0.123 (0.082, 0.166)	0.117	0.343	0.669	0.649
Barn swallow - Badajoz	62.97 (49.32, 72.15)	0.159 (0.11, 0.23)	0.178	0.186	0.898	0.356
Barn swallow - Kraghede	63.38 (46.33, 74.2)	0.222 (0.122, 0.335)	0.251	0.309	0.916	0.054
Blue tit - Muro	64.56 (48.99, 70.71)	0.107 (0.069, 0.153)	0.185	0.388	0.752	0.499
Blue tit - Pirio	64.7 (55.52, 73.93)	0.111 (0.073, 0.143)	0.188	0.474	0.814	0.279
Blue tit - Rouvière	59.38 (52.12, 65.7)	0.127 (0.101, 0.16)	0.174	0.274	0.864	0.384
Collared flycatcher	59.5 (55.18, 64.45)	0.131 (0.111, 0.151)	0.154	0.304	0.919	0.2
Savannah sparrow	62.24 (49.06, 68.75)	0.163 (0.11, 0.217)	0.145	0.226	0.8	0.537
House sparrow	73.65 (60.72, 80.16)	0.256 (0.171, 0.383)	0.194	0.494	0.764	0.366

### Natural selection on morphology

Both the direction and strength of selection varied substantially across species but also across populations. In four of the populations (the three blue tit populations and the Kraghede population of barn swallows), directional selection was significant on bill length, tarsus or mass, but not on wing length (Tables 7, 8 and 9). In collared flycatchers, great reed warblers and Savannah sparrows (Tables 7 and 9), we found significant directional selection on two traits, and in these three cases, selection was negative on mass and positive either on wing, tarsus or bill length, respectively. We also found no evidence of significant nonlinear selection. There was evidence for negative correlated selection on tarsus and mass in blue tits (Pirio) and barn swallows (Kraghede) and on tarsus and wing in barn swallows (Badajoz). Finally, there was significant positive correlated selection on bill length and wing in blue tits (Pirio), and wing length and mass in house sparrow. No significant selection was found in red-billed gulls.

**Table 7:** Estimates of directional and non-linear selection gradients for the Red-billed gull, Great reed warbler, and the two Barn swallow populations with their 95% confidence intervals.

	Red-billed gull			Great reed warbler			Barn swallow - Badajoz			Barn swallow - Kraghede		
	posterior mode	Low 95%CI	Up 95%CI	posterior mode	Low 95%CI	Up 95%CI	posterior mode	Low 95%CI	Up 95%CI	posterior mode	Low 95%CI	Up 95%CI
|| β ||	4.22	1.94	7.24	5.23	2.53	8.17	0.54	0.22	1.48	1.06	0.4	2.02
Wing	1.14	−2.75	5.9	−0.09	−3.79	4.12	0.33	−0.72	1.5	−0.54	−1.89	1.23
Tarsus	1.86	−1.12	4.39	**4.04**	**1.45**	**7.69**	0.12	−0.48	0.65	**0.7**	**0.09**	**1.29**
Mass	−1.15	−2.39	0.32	**−1.78**	**−3.02**	**−0.51**	0.08	−0.27	0.44	−0.17	−0.48	0.4
Bill	1.86	−1.83	5.35	0.58	−1.48	1.96	0.15	−0.25	0.59	0.32	−0.27	0.86
Wing^2^	139.96	−27.15	243.35	15.61	−107.1	233.37	−13.47	−35.27	7.07	41.28	−15	91.45
Tarsus^2^	29.1	−33.29	88.21	24.88	−61.64	128.76	2.78	−5.35	9.58	1	−5.6	11.14
Mass^2^	9.26	−8.08	21.38	−0.57	−18.42	12.75	−1.71	−5.6	1.44	2.13	−1.94	5.75
Bill^2^	−57.69	−151.95	50.09	22.47	−4.12	51.95	−0.93	−5.75	2.37	4.99	−2.93	11.9
Wing:Tarsus	−108.46	−274.15	28.38	−50.82	−212.11	160.11	**−22.4**	**−49.61**	**−0.74**	11.18	−13.68	40.7
Wing:Mass	18.4	−56.76	79.17	−41.18	−114.47	45.77	14.48	−3.85	24.46	10.04	−10.91	32.6
Tarsus:Mass	−3.22	−53.47	43.52	30.94	−20.81	84.41	10.08	−0.61	15.37	**−10.96**	**−20.1**	**−0.71**
Mass:Bill	−10.09	−62.84	50.76	4.29	−27.27	29.81	3.25	−2.06	9.56	−1.65	−9.87	6.87
Tarsus:Bill	−30.88	−127.8	97.93	−39.99	−132.73	20.27	−4.47	−11.57	5.86	−2.85	−15.28	7
Wing:Bill	−56.3	−273.35	124.82	12.16	−109.53	89.27	14.36	−8.2	25.07	2.55	−20.86	30.12

In bold are the estimates significantly different from zero. || β || is the norm of the directional selection gradient. Note that the quadratic coefficients are not doubled in this table.

**Table 8:** Estimates of directional and non-linear selection gradients for the three Blue tit populations with their 95% confidence intervals.

	Blue tit - Muro			Blue tit - Pirio			Blue tit - Rouvière		
	posterior mode	Lower 95%CI	Upper 95%CI	posterior mode	Lower 95%CI	Upper 95%CI	posterior mode	Lower 95%CI	Upper 95%CI
|| β ||	0.97	0.41	1.38	1.31	0.67	1.95	1.08	0.48	1.66
Wing	0.24	−0.53	0.91	0.46	−0.4	1.53	0.23	−0.31	1.06
Tarsus	0.48	−0.22	1.06	0.42	−0.27	1.39	**0.91**	**0.3**	**1.55**
Mass	−0.29	−0.69	0.09	**−0.82**	**−1.27**	**−0.39**	−0.22	−0.62	0.13
Bill	**0.63**	**0.13**	**0.88**	0.39	−0.18	0.86	0.05	−0.29	0.46
Wing^2^	3.43	−14.12	18.72	−15.15	−34.66	13.41	−2.15	−18.01	6.11
Tarsus^2^	2.75	−13.17	14.73	13.96	−4.41	40.91	−6.73	−24.06	15.68
Mass^2^	−1.56	−5.86	3.06	6.16	−0.63	11.42	−2.05	−6.77	2.83
Bill^2^	−1.8	−8.19	4.09	−3.55	−11.84	2.91	2.44	−3.61	7.67
Wing:Tarsus	−25.07	−43.24	10.21	−21.05	−55.97	22.68	2.72	−26.45	27.8
Wing:Mass	6.84	−11.44	22.93	11.87	−11.67	32.68	−4.46	−16.57	15.48
Tarsus:Mass	9.81	−2.19	25.34	**−27.21**	**−46.44**	**−6.3**	−2.9	−15.78	12.68
Mass:Bill	−1.77	−9.21	8.08	−0.45	−7.34	10.49	−4.38	−10.7	5.79
Tarsus:Bill	8.74	−8.22	20.52	−18.93	−38.82	2.03	−10.73	−25.35	4.48
Wing:Bill	4.59	−10.91	23.65	**28.53**	**2.35**	**51.24**	2.23	−16.82	17.16

In bold are the estimates significantly different from zero. || β || is the norm of the directional selection gradient. Note that the quadratic coefficients are not doubled in this table.

**Table 9:** Estimates of directional and non-linear selection gradients for the Collared flycatcher, Savannah sparrow and House sparrow populations with their 95% confidence intervals.

	Collared flycatcher			Savannah sparrow			House sparrow		
	posterior mode	Lower 95%CI	Upper 95%CI	posterior mode	Lower 95%CI	Upper 95%CI	posterior mode	Lower 95%CI	Upper 95%CI
|| β ||	0.93	0.57	1.53	2.34	1.33	3.49	1.91	0.74	3.67
Wing	**0.74**	**0.12**	**1.33**	0.94	−1.13	2.4	−0.42	−2.7	2.56
Tarsus	0.52	−0.01	0.96	−1.32	−2.39	0.37	1.16	−0.26	2.43
Mass	**−0.39**	**−0.59**	**−0.21**	**−0.73**	**−1.27**	**−0.12**	0.05	−1.09	0.61
Bill	−0.11	−0.47	0.18	**1.73**	**0.86**	**2.49**	−0.87	−1.85	0.54
Wing^2^	7.64	−9.72	29.03	−35.23	−89.95	21.92	3.24	−118.06	80.24
Tarsus^2^	−1.4	−11.48	5.68	0.47	−41.13	42.94	−4.57	−40.03	26.08
Mass^2^	−1.18	−2.98	0.32	−5.13	−11.24	0.09	−7.91	−18.66	2.23
Bill^2^	0.88	−2.82	4.51	8.49	−2.38	26.02	19.05	−4.77	37.6
Wing:Tarsus	−10.88	−31.36	14.78	−45.03	−105.1	21.11	−57.39	−163.83	12.26
Wing:Mass	−3.71	−11.99	7.59	26.43	−2.21	62.14	**76.43**	**35.36**	**128.3**
Tarsus:Mass	4.57	−1.07	13.75	2.37	−25.51	24.2	10.74	−19.19	39.52
Mass:Bill	−2.84	−7.46	2.59	4.2	−12.41	14.79	−11.88	−38.77	15.99
Tarsus:Bill	−6.85	−21.58	5.01	−2.72	−33.07	32.1	25.05	−15.52	55.11
Wing:Bill	7.42	−10.11	21.37	−4.27	−48.02	33.75	−36	−114.1	31.27

In bold are the estimates significantly different from zero. || β || is the norm of the directional selection gradient. Note that the quadratic coefficients are not doubled in this table.

### Constraints on predicted responses to current selection

**Figure 2:**
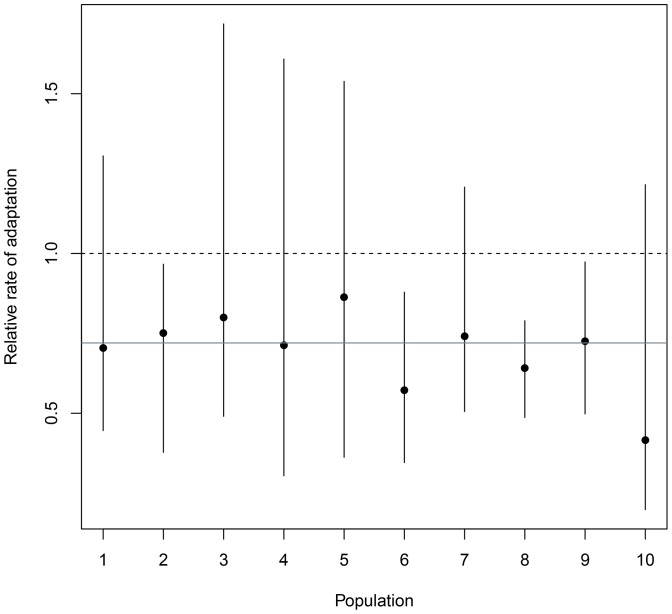
Relative rate of adaptation (R_A_) in the 10 populations. Dots represent posterior mode estimates and lines the 95% confidence interval. The dotted black line at 0.75 represents the geometric mean of all populations while the dotted grey line at 1 shows the case of no effect of genetic correlations. Population number refers to the numbers given in Table 1.

The predicted rate of adaptation was significantly lower in the presence than in the absence of genetic correlations (i.e., 95% of R_A_ values from the posterior distribution lower than 1) in four of the 10 populations (Table 10, Fig. 2): great reed warblers, blue tits in Pirio, collared flycatchers and Savannah sparrows. On average, R_A_ was 72%, which means that because of genetic correlations, the predicted fitness gain was on average 28% lower than it would be in the absence of these correlations. Despite large confidence intervals around the geometric mean across all populations, this average decrease was significant (geometric mean with 95% CI: 0.72 (0.60, 0.85), Fig. 2), and no R_A_ was larger than 1.

**Table 10:** Rate of adaptation, evolvability and orientation of genetic variance relative to selection gradients (β) for ten bird populations.

		Rate of adaptation (R_A_)				Multivariate evolvability (e_β_)			Average evolvability ( 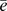 )			θ_gmax_ - Angle between β and g_max_		
Species	Population	Estimate	Lower CI	Higher CI	PS(R_A_<1)	Estimate	Lower CI	Higher CI	Estimate	Lower CI	Higher CI	Estimate	Lower CI	Higher CI
Red billed gull	1 - Kaikoura, New Zealand	0.704	0.446	1.305	83.5	0.0349	0.0208	0.0533	0.0554	0.0498	0.0644	86.26	65.20	89.97
Great reed warbler	2 - Kvismaren, Sweden	**0.751**	**0.378**	**0.966**	**97.3**	0.0241	0.0143	0.0425	0.0578	0.0436	0.0684	87.86	68.09	89.98
Barn swallow	3 - Badajoz, Spain	0.800	0.491	1.718	45.7	0.0267	0.0082	0.1094	0.0639	0.0499	0.0853	79.14	41.42	90.00
	4 - Kraghede, Denmark	0.713	0.305	1.608	67.3	0.0528	0.0127	0.1241	0.0896	0.0611	0.1225	87.74	50.22	90.00
Blue tit	5 - Muro, France	0.864	0.362	1.538	71.2	0.0306	0.0144	0.0633	0.0446	0.0338	0.0567	81.07	49.49	90.00
	6 - Pirio, France	**0.572**	**0.346**	**0.879**	**98.1**	0.0257	0.0111	0.0503	0.0416	0.0320	0.0505	86.89	56.73	89.95
	7 - Rouviere, France	0.741	0.506	1.208	85.7	0.0380	0.0207	0.0512	0.052	0.0455	0.0627	89.22	65.15	89.97
Collared flycatcher	8 - Gotland, Sweden	**0.642**	**0.487**	**0.789**	**100.0**	0.0239	0.0149	0.0411	0.0545	0.0489	0.0602	87.84	66.01	89.99
Savannah sparrow	9 - Kent Island, Canada	**0.726**	**0.498**	**0.973**	**98.4**	0.0477	0.0255	0.0746	0.0663	0.0536	0.0829	86.18	70.17	89.98
House sparrow	10 - Lundy, UK	0.416	0.199	1.215	89.4	0.0351	0.0108	0.0928	0.0937	0.0686	0.1254	85.24	61.97	90.00

The relative rate of adaptation R_A_ is calculated according to eq (2) and compares the rate of adaptation in the presence and the absence of genetic correlations. If the rate of adaptation is lower than 1, genetic correlations slow down adaptation. For each population the proportion of support of the posterior distribution (PS) for the hypothesis of R_A_<1 is also given. In bold are shown significant estimates where the posterior distribution supports the hypothesis by at least 95%. Multivariate evolvability (×100), the amount of predicted response occurring exactly in the direction of selection was calculated according to eq (4). Average evolvability (×100), the average evolvability in random direction of phenotypic space was calculated according to eq (5). The angles between selection gradients and g_max_ (θ_gmax_) were calculated according to eq (6).

**Figure 3:**
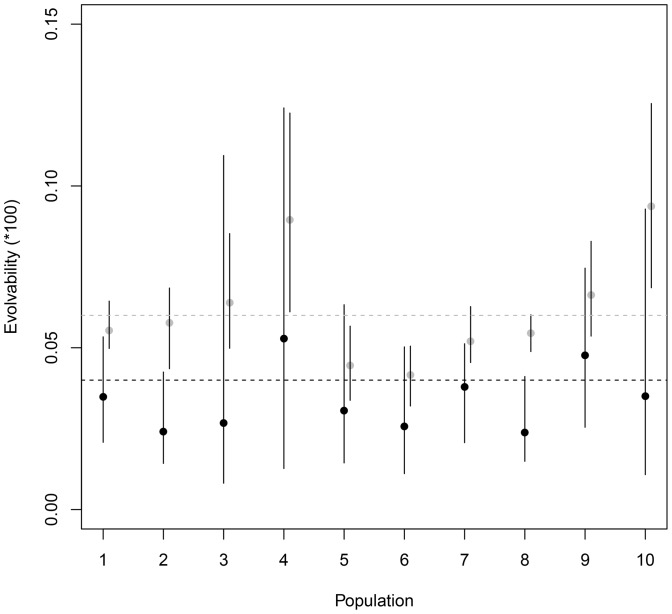
Comparison of evolvabilities in the direction of selection (e_β_, black symbols) and average evolvabilities in random directions of phenotypic space (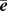 **, grey symbols).** Dotted lines represent the average value over the ten populations. Population number refers to the numbers given in Table 1.

Evolvability in the direction of **β** was on average 1.7 times lower than in random directions (mean e_β_ ×100 (95% CI): 0.0369 (0.0291; 0.0509), mean 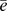 ×100 (95% CI) : 0.0638 (0.0577; 0.0680), Table 10, Fig. 3), implying that current selection is acting in a direction of lower genetic variance than the average genetic variation in the phenotypic space. Confidence intervals within populations are much larger for e_β_ than for 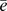 due to the uncertainty in the **β** estimates which adds to the uncertainty on **G** estimates. In accordance with these results on evolvability, the vectors **g_max_** and **β** were very close to orthogonal in most populations (Table 10), so that if all genetic variance was along **g_max_**, no response to selection would be possible. However, other dimensions of phenotypic space include 40% of genetic variance, so that multivariate evolvabilities (e_β_, the amount of response in the exact direction of **β**) were significantly different from zero (Table 10). These results emphasise that genetic variance remaining along dimensions other than **g_max_** also play a major role in determining evolvabilities.

## Discussion

We report consistent evidence for multivariate constraints on morphological evolution across 10 avian populations studied in their natural habitat during extensive periods exceeding 12 years. Morphological traits generally display high heritabilities and harbour ample additive genetic variation [34], [35], [48]–[50]. Therefore they are often believed to be only weakly constrained in terms of evolutionary potential but see [40], [51]. Here for linear measurements (mass excluded) we found I_A_-evolvabilities less than half (0.04% on average) of what was reported (0.09%) in the review by Hansen et al. [40]. The highest I_A_-evolvabilities were found for body mass, yet again for this trait, our estimates of I_A_-evolvability were much lower (0.12%) than the previously reported average of 0.94% [40]. In a univariate framework, for a trait with an I_A_-evolvability of 0.04%, this means that, if selection acting on this trait was as strong as on fitness itself, a change of 10% in the mean of the trait would be achieved in 240 generations [40].

Moreover, using a multivariate framework, we also found evidence of evolutionary constraints even when only four morphological traits were considered, emphasising that equating heritability with evolutionary potential can be misleading [40]. In fact, here we have shown that the predicted relative rate of adaptation (R_A_) was on average 72%, which means that the predicted rate of adaptation was lowered by 28% (1- R_A_, range 13–58%) due to the genetic correlations considered.

Two scenarios may lead to a decreased rate of adaptation: negative genetic correlations with similar direction of selection pressures or positive genetic correlations in the presence of antagonistic selection. Negative genetic correlations have gained much interest in the study of evolutionary constraints [4]. This is mainly because selection is often positive on life history traits so that trade-offs should emerge as a consequence of negative genetic correlations for these traits but see [52]. However, genetic correlations between morphological traits generally seem to be positive ([53], this study, review in [54]). As the sign of selection on morphological traits is not always positive but depends on traits and populations ([49], this study), opposing selection patterns within the same organisms can be common and hence lead to constraints on responses to selection. Here, this scenario is illustrated by three populations of great reed warblers, collared flycatchers and Savannah sparrows, where the relative rate of adaptation was significantly lower than one. In these populations, antagonistic selection between mass and another trait (tarsus, wing and bill length, respectively), in the presence of positive genetic correlations explain this result. Such opposing selection patterns can arise because of selection for a specific function. For example, selection on wing length can be positive or negative, depending on whether long-distance flight or manoeuvrability are favoured e.g. [55]. Similarly, the sign of selection on beak size in Darwin's finches (*Geospiza fortis*) depends on the abundance of different seed types, which themselves depend on climatic events [56]. Further studies in each population would be needed to interpret selection patterns in terms of the function of traits, and to assess the ecological determinants behind these patterns.

Such a reduction in the rate of adaptation reflects changes between the predicted responses to selection of traits whether or not genetic correlations are taken into account. In great reed warblers, univariate models (i.e., not taking into account genetic correlations) predict significant responses in tarsus length and mass to selection, but no significant response in either trait is expected in the presence of genetic correlations (Table S3). In collared flycatchers univariate models predict a response to selection in both wing length and mass, but multivariate models predict a significant response only in mass. In Savannah sparrows, univariate models predict a response to selection in both mass and bill length, but only bill length is predicted to respond to selection in the presence of genetic correlations (Table S3). In contrast, no significant antagonistic selection was found in blue tits in Pirio, but multivariate models reveal nonetheless a disappearance of the response in mass when compared to univariate models. This is probably due to the fact that selection is significantly negative on mass while although non-significant, it is positive on the three other traits.

We found a consistent pattern in that the orientation of **g_max_** was nearly orthogonal to directional selection in all populations. Although **g_max_** contained on average 60% of the additive genetic variance, the dimensions of **G** other than **g_max_** still contained ca. 40% of additive genetic variance. This suggests that genetic correlations can decrease the rate of adaptation, but do not necessarily lead to an absolute constraint (i.e., here R_A_≠0). It is thus important to consider other dimensions along which additive genetic variance is distributed, and not only **g_max_** [19], [57], as a reduction of the rate of adaptation of 28% is lower than what could have been expected based on the relative orientation of selection and **g_max_**.

In line with this argument, evolvability in the direction of selection (e_β_) was on average lower than evolvability in random directions of the phenotypic space (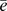), suggesting that selection may have reduced available genetic variance. This may be a very general pattern: depleted genetic variance in the direction of selection has also been found in sexually selected traits [20], [58], [59] and life history traits [60].This result could suggest a depletion of additive genetic variance because of sustained directional selection on particular trait combinations [4]. However, there is still a debate about the stability of selection [61], [62], so that a spatiotemporal analysis of selection patterns in each population would be required to assess whether sustained selection can be responsible for the observed pattern. Evolvabilities from this study (either e_β_ or 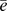) are very low compared to estimates from Simonsen and Stinchcombe [60] on life history traits of the ivyleaf morning glory (*Ipomoea hederacea*, e_β_∼0.002) or foraging traits of three-spined sticklebacks (*Gasterosteus aculeatus*, e_β_∼0.015 and 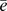  = 0.007, [19]). However, results are similar to what was found by Björklund et al [63] in the same collared flycatcher population that we studied. There are still very few studies reporting estimates of multivariate evolvabilities, and it is not possible yet to interpret these differences either in terms of traits or taxa, yet we hope that our results will encourage further estimates in the near future.

While our estimate of a decrease in predicted rate of adaptation (28%) is 2.5 times as large as the average decrease estimated by Agrawal & Stinchcombe [13] in their review (11%), they also found in 12 out of 45 studies that genetic correlations decreased the rate of adaptation by more than 30%. The decrease in predicted rate of adaptation from the present study is also lower than that found by Morrissey et al. [14] in their study of life history traits in a single island population of red deer (*Cervus elaphus*, 40%). In the Spanish population (Badajoz) of barn swallows, Teplitsky et al. [15] found a decrease of 48% of the rate of adaptation for life history traits whereas in the present study we found a (non-significant) decrease of 20% for morphological traits for the same population. Two main factors may help explain such differences across studies. First, it is possible that morphological and life history traits differ in the amount of genetic constraints. Second, if selection is stable, constraints might actually be detected more readily than facilitation in natural populations. If genetic correlations facilitate the response to selection, populations should adapt and be subject to less intense selection. Hence, facilitation could be a transient state whereas constraints would represent a more stable state. Further analysis of data, such as those gathered in Agrawal & Stinchcombe [13], could provide valuable information as to when facilitation is more likely to occur. For example, does facilitation occur when organisms are subject to recent selection pressures, or when genetic architecture changes under new environmental conditions?

The existence of multivariate constraints can have important implications for the potential of a micro-evolutionary response to rapid changes in the environment such as global climate change, because the pace of microevolution may be considerably reduced. The prevalence of such genetic constraints may begin to explain why so far little evidence of evolutionary adaptation to climate change has been reported [64]. The evolutionary significance of these constraints will also depend on the stability of the **G** matrix. The discussion regarding the extent to which and the conditions under which **G** is stable is still open, as some studies revealed either surprising constancy of **G** (review in [65], [66]) or rapid changes [63].

Finally, our study showed significant multivariate constraints even though only four traits were included. This represents a very small fraction of all the traits integrated within an organism, and it is likely that constraints would become stronger if more traits were included [67]. As evidence is building that including more traits dramatically affects predicted responses to selection (e.g. [68], Table S3), and as our understanding and appreciation of evolutionary trajectories improves, it is becoming clear that multivariate studies should be the standard approach in evolutionary biology. Of course, including all traits is unachievable, but more comprehensive approaches based, for example, on modularity and identified suites of functionally related and highly correlated characters relatively independent of other suites of traits [69], promise to bring significant insights.

### Conclusions

Our study assesses the generality of evolutionary constraints on morphology in birds that may arise from selection pressures such as those due to rapid environmental change. We found multivariate constraints on the predicted response to selection in morphological traits. Such traits are generally thought of as having a high evolutionary potential, which highlights the danger of equating heritability and evolutionary potential, as this can lead to an overestimation of the rate of adaptation. This can be especially problematic when assessing the sustainable rate of environmental change above which adaptation will be too slow to prevent population extinction [70].

## Supporting Information

Figure S1 **Histograms of relatedness between pairs of individuals present in the pruned pedigree for each of the populations.**
(DOC)

Figure S2
**Graphical comparison of the estimates of rate of adaptation, multivariate evolvability (e_β_), average evolvability (** 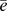 **) and angle between gmax and directional selection using slightly informative prior and parameter expanded prior.**
(DOC)

Table S1 **Significance of fixed effects in final models after removal of non-significant effects.**
(DOC)

Table S2 **Estimates of heritabilities (with traits standardized to the variance) for each population.**
(DOC)

Table S3 **Predicted responses to selection (×100) in multivariate and univariate frameworks, and the angle between selection and predicted response to selection (Angle(R, β)).**
(DOC)
