# Supplementary material for: Assessing Multivariate Constraints to Evolution across Ten Long-Term Avian Studies
Source: PLoS One. 2014 Mar 7;9(3):e90444. doi: 10.1371/journal.pone.0090444 (PMC3946496; doi:10.1371/journal.pone.0090444)

**Figure S1**: Histograms of relatedness between pairs of individuals present in the pruned pedigree for each of the populations (black line: number of pairs for each level of relatedness, grey dot: no pair was found at this level).


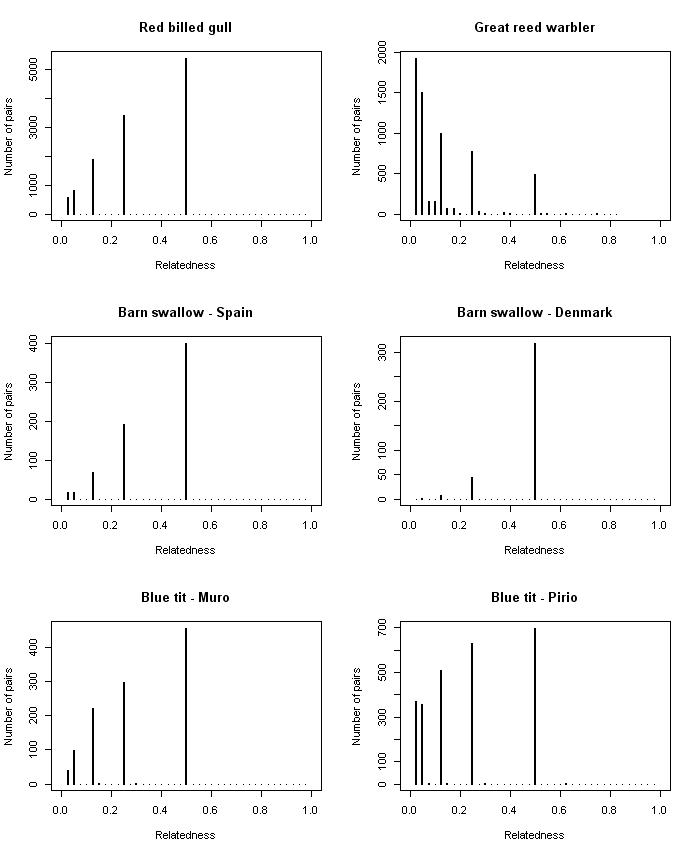


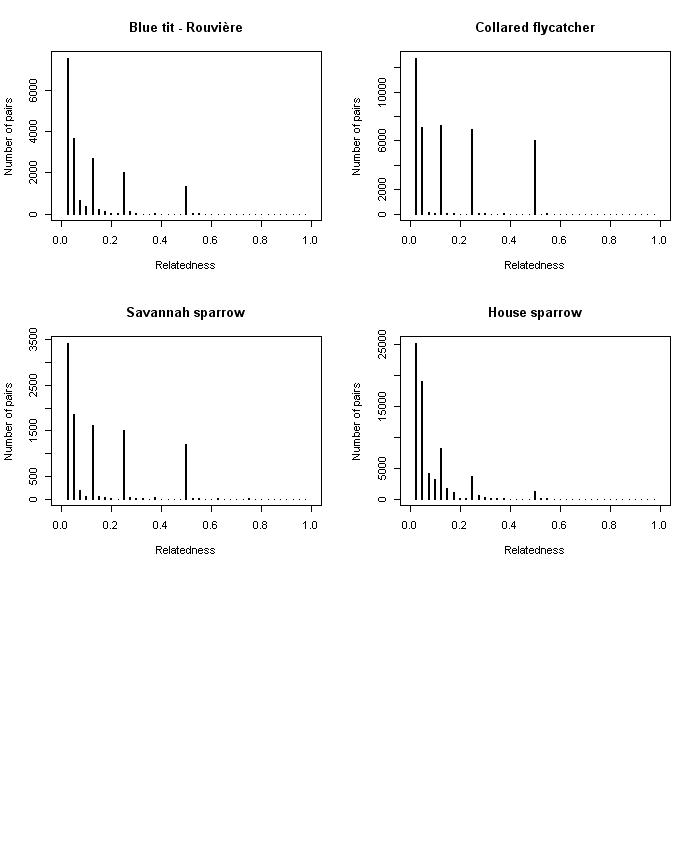

Supplement: Figure S1 — Histograms of relatedness between pairs of individuals present in the pruned pedigree for each of the populations. (DOC) [file pone.0090444.s001.doc]
