# Supplementary material for: Assessing Multivariate Constraints to Evolution across Ten Long-Term Avian Studies
Source: PLoS One. 2014 Mar 7;9(3):e90444. doi: 10.1371/journal.pone.0090444 (PMC3946496; doi:10.1371/journal.pone.0090444)
Supplement: Figure S2 — Graphical comparison of the estimates of rate of adaptation, multivariate evolvability (eβ), average evolvability ( ) and angle between gmax and directional selection using slightly informative prior and parameter expanded prior. (DOC) [file pone.0090444.s002.doc]

**Figure S2:** Graphical comparison of the estimates of rate of adaptation (a), multivariate evolvability (e_β_, b), average evolvability ($\bar{e}$, c) and angle between gmax and directional selection (d) using slightly informative prior (black) and parameter expanded prior (grey). The white dots represent the values obtained based on posterior modes of **G** matrices with the slightly informative prior and posterior modes of selection estimates. The largest differences are found in the Danish and Spanish barn swallow populations for which the power of the statistical test is the lowest. Numbers on x-axis refer to populations in Table 1. Dots are posterior modes of the variable and the bars represent the 95% confidence interval. See Materials and Methods for further details.
