# Supplementary material for: Assessing Multivariate Constraints to Evolution across Ten Long-Term Avian Studies
Source: PLoS One. 2014 Mar 7;9(3):e90444. doi: 10.1371/journal.pone.0090444 (PMC3946496; doi:10.1371/journal.pone.0090444)
Supplement: Table S1 — Significance of fixed effects in final models after removal of non-significant effects. (DOC) [file pone.0090444.s003.doc]

**Table S1:** Significance of fixed effects in final models after removal of non-significant effects. Traits with a P value less than or equal to 0.1 were kept in the final models to be conservative in selecting models. In grey are shown effects not included in the models.

1. Red-billed gull

|  | Wing length | | | | Tarsus length | | | | Mass | | | | Bill length | | | |
| --- | --- | --- | --- | --- | --- | --- | --- | --- | --- | --- | --- | --- | --- | --- | --- | --- |
|  | estimate | Lower CI | Upper CI | P | estimate | Lower CI | Upper CI | P | estimate | Lower CI | Upper CI | P | estimate | Lower CI | Upper CI | P |
| Intercept | 32.563 | 32.466 | 32.660 | <0.001 | 24.071 | 23.942 | 24.210 | <0.001 | 11.413 | 11.319 | 11.507 | <0.001 | 22.576 | 22.518 | 22.639 | <0.001 |
| Age | 0.037 | 0.029 | 0.046 | <0.001 | -0.015 | -0.027 | -0.003 | 0.018 | 0.005 | -0.004 | 0.014 | 0.310 | 0.010 | 0.006 | 0.014 | <0.001 |
| Age² | -0.003 | -0.004 | -0.002 | <0.001 | 0.000 | -0.001 | 0.002 | 0.736 | -0.001 | -0.002 | 0.000 | 0.028 | -0.001 | -0.001 | 0.000 | 0.002 |
| Sex | -1.381 | -1.441 | -1.314 | <0.001 | -1.173 | -1.259 | -1.074 | <0.001 | -1.180 | -1.228 | -1.133 | <0.001 | -1.696 | -1.728 | -1.659 | <0.001 |
| Age:Sex | -0.008 | -0.021 | 0.003 | 0.216 | 0.015 | -0.002 | 0.029 | 0.068 | 0.018 | 0.006 | 0.031 | 0.004 | -0.012 | -0.017 | -0.006 | <0.001 |
| Age²:Sex | 0.000 | -0.001 | 0.002 | 0.548 | 0.000 | -0.002 | 0.002 | 0.748 | -0.001 | -0.002 | 0.001 | 0.362 | 0.001 | 0.000 | 0.002 | <0.001 |

1. Great reed warbler

|  | Wing length | | | | Tarsus length | | | | Mass | | | | Bill length | | | |
| --- | --- | --- | --- | --- | --- | --- | --- | --- | --- | --- | --- | --- | --- | --- | --- | --- |
|  | estimate | Lower CI | Upper CI | P | estimate | Lower CI | Upper CI | P | estimate | Lower CI | Upper CI | P | estimate | Lower CI | Upper CI | P |
| Intercept | 36.421 | 36.156 | 36.577 | 0.000 | 34.150 | 33.898 | 34.459 | 0.000 | 14.897 | 14.622 | 15.227 | 0.000 | 22.660 | 22.282 | 23.056 | 0.000 |
| Age | 0.195 | 0.120 | 0.279 | 0.000 | 0.044 | -0.018 | 0.151 | 0.148 | 0.299 | 0.137 | 0.443 | 0.000 | -0.196 | -0.376 | 0.005 | 0.066 |
| Age² | -0.063 | -0.084 | -0.028 | 0.000 | -0.006 | -0.035 | 0.027 | 0.702 | -0.046 | -0.106 | 0.005 | 0.062 | 0.046 | -0.039 | 0.113 | 0.452 |
| Sex | -1.467 | -1.570 | -1.352 | 0.000 | -1.267 | -1.393 | -1.103 | 0.000 | -0.720 | -0.892 | -0.537 | 0.000 | -0.305 | -0.525 | -0.107 | 0.006 |
| Age:Sex | -0.019 | -0.061 | 0.034 | 0.540 | -0.041 | -0.101 | 0.002 | 0.072 | -0.218 | -0.342 | -0.155 | 0.000 | 0.117 | -0.007 | 0.222 | 0.086 |
| Age²:Sex | 0.007 | -0.004 | 0.028 | 0.128 | 0.002 | -0.011 | 0.024 | 0.468 | 0.044 | 0.011 | 0.076 | 0.002 | -0.014 | -0.053 | 0.032 | 0.580 |
| Day |  |  |  |  |  |  |  |  | 0.025 | 0.007 | 0.040 | 0.010 |  |  |  |  |
| Day² |  |  |  |  |  |  |  |  | 0.000 | 0.000 | 0.001 | 0.628 |  |  |  |  |
| Day^3^ |  |  |  |  |  |  |  |  | 0.000 | 0.000 | 0.000 | 0.006 |  |  |  |  |
| Day:Sex |  |  |  |  |  |  |  |  | -0.030 | -0.040 | -0.019 | 0.000 |  |  |  |  |
| Day²:Sex |  |  |  |  |  |  |  |  | 0.000 | -0.001 | 0.000 | 0.452 |  |  |  |  |
| Day^3^:Sex |  |  |  |  |  |  |  |  | 0.000 | 0.000 | 0.000 | 0.004 |  |  |  |  |

1. Barn swallow – Spain

|  | Wing length | | | | Tarsus length | | | | Mass | | | | Bill length | | | |
| --- | --- | --- | --- | --- | --- | --- | --- | --- | --- | --- | --- | --- | --- | --- | --- | --- |
|  | estimate | Lower CI | Upper CI | P | estimate | Lower CI | Upper CI | P | estimate | Lower CI | Upper CI | P | estimate | Lower CI | Upper CI | P |
| Intercept | 39.720 | 39.346 | 40.047 | <0.001 | 20.851 | 20.385 | 21.277 | <0.001 | 10.816 | 10.360 | 11.223 | <0.001 | 13.965 | 13.516 | 14.405 | <0.001 |
| Age | 0.136 | -0.112 | 0.402 | 0.304 | -0.016 | -0.397 | 0.349 | 0.922 | 0.708 | 0.392 | 1.043 | <0.001 | -0.101 | -0.441 | 0.231 | 0.622 |
| Age² | -0.009 | -0.055 | 0.036 | 0.682 | -0.006 | -0.072 | 0.064 | 0.884 | -0.110 | -0.175 | -0.056 | <0.001 | 0.005 | -0.054 | 0.067 | 0.876 |
| Sex | -0.546 | -0.724 | -0.342 | <0.001 | 0.016 | -0.218 | 0.288 | 0.91 | 0.443 | 0.194 | 0.659 | 0.002 | -0.184 | -0.418 | 0.061 | 0.15 |
| Age:Sex | 0.044 | -0.127 | 0.203 | 0.592 | 0.092 | -0.141 | 0.355 | 0.454 | -0.299 | -0.524 | -0.095 | 0.008 | 0.108 | -0.105 | 0.330 | 0.358 |
| Age²:Sex | -0.016 | -0.045 | 0.014 | 0.34 | -0.010 | -0.053 | 0.037 | 0.65 | 0.051 | 0.013 | 0.090 | 0.012 | -0.012 | -0.052 | 0.025 | 0.63 |

1. Barn swallow – Denmark

|  | Wing length | | | | Tarsus length | | | | Mass | | | | Bill length | | | |
| --- | --- | --- | --- | --- | --- | --- | --- | --- | --- | --- | --- | --- | --- | --- | --- | --- |
|  | estimate | Lower CI | Upper CI | P | estimate | Lower CI | Upper CI | P | estimate | Lower CI | Upper CI | P | estimate | Lower CI | Upper CI | P |
| Intercept | 42.961 | 42.649 | 43.255 | <0.001 | 17.015 | 16.718 | 17.356 | <0.001 | 12.106 | 11.775 | 12.420 | <0.001 | 15.615 | 15.321 | 15.927 | <0.001 |
| Age | -0.223 | -0.930 | 0.452 | 0.522 | -0.130 | -1.064 | 0.751 | 0.762 | -0.075 | -0.841 | 0.720 | 0.828 | 0.783 | -0.038 | 1.530 | 0.048 |
| Age² | 0.064 | -0.235 | 0.382 | 0.678 | 0.102 | -0.335 | 0.484 | 0.606 | -0.035 | -0.391 | 0.304 | 0.854 | -0.451 | -0.805 | -0.107 | 0.008 |
| Sex | -0.467 | -0.637 | -0.287 | <0.001 | -0.036 | -0.196 | 0.141 | 0.712 | 0.466 | 0.300 | 0.659 | <0.001 | 0.017 | -0.157 | 0.193 | 0.83 |
| Age:Sex | 0.340 | -0.118 | 0.793 | 0.132 | 0.032 | -0.536 | 0.611 | 0.882 | 0.158 | -0.320 | 0.681 | 0.546 | -0.433 | -0.928 | 0.065 | 0.098 |
| Age²:Sex | -0.084 | -0.272 | 0.097 | 0.376 | -0.050 | -0.292 | 0.176 | 0.628 | -0.016 | -0.209 | 0.188 | 0.832 | 0.244 | 0.039 | 0.429 | 0.016 |

1. Blue tit – Muro

|  | Wing length | | | | Tarsus length | | | | Mass | | | | Bill length | | | |
| --- | --- | --- | --- | --- | --- | --- | --- | --- | --- | --- | --- | --- | --- | --- | --- | --- |
|  | estimate | Lower CI | Upper CI | P | estimate | Lower CI | Upper CI | P | estimate | Lower CI | Upper CI | P | estimate | Lower CI | Upper CI | P |
| Intercept | 32.720 | 32.500 | 32.970 | <0.001 | 33.240 | 32.980 | 33.490 | <0.001 | 20.880 | 20.580 | 21.190 | <0.001 | 19.390 | 19.040 | 19.710 | <0.001 |
| Age | 0.733 | 0.563 | 0.887 | <0.001 | 0.020 | -0.132 | 0.181 | 0.798 | 0.139 | -0.071 | 0.338 | 0.200 | -0.073 | -0.283 | 0.152 | 0.514 |
| Age² | -0.253 | -0.325 | -0.179 | <0.001 | 0.017 | -0.053 | 0.085 | 0.650 | -0.013 | -0.114 | 0.092 | 0.796 | 0.019 | -0.088 | 0.132 | 0.738 |
| Sex | -1.260 | -1.372 | -1.161 | <0.001 | -0.866 | -0.983 | -0.738 | <0.001 | -0.373 | -0.514 | -0.224 | <0.001 | 0.536 | 0.397 | 0.670 | <0.001 |
| Age:Sex | -0.177 | -0.273 | -0.071 | <0.001 | -0.030 | -0.125 | 0.061 | 0.530 | -0.055 | -0.172 | 0.068 | 0.410 | 0.118 | -0.017 | 0.245 | 0.086 |
| Age²:Sex | 0.097 | 0.051 | 0.138 | <0.001 | -0.008 | -0.047 | 0.034 | 0.712 | -0.001 | -0.061 | 0.056 | 0.966 | -0.043 | -0.109 | 0.016 | 0.186 |
| Day |  |  |  |  |  |  |  |  | -0.001 | -0.026 | 0.021 | 0.954 | -0.010 | -0.033 | 0.010 | 0.404 |
| Day² |  |  |  |  |  |  |  |  | 0.001 | 0.000 | 0.002 | 0.228 | 0.000 | 0.000 | 0.001 | 0.458 |
| Day^3^ |  |  |  |  |  |  |  |  | 0.000 | 0.000 | 0.000 | 0.114 | 0.000 | 0.000 | 0.000 | 0.762 |
| Day:Sex |  |  |  |  |  |  |  |  | -0.015 | -0.029 | -0.002 | 0.034 | -0.001 | -0.015 | 0.012 | 0.896 |
| Day²:Sex |  |  |  |  |  |  |  |  | 0.000 | -0.001 | 0.001 | 0.790 | 0.000 | -0.001 | 0.000 | 0.564 |
| Day^3^:Sex |  |  |  |  |  |  |  |  | 0.000 | 0.000 | 0.000 | 0.084 | 0.000 | 0.000 | 0.000 | 0.284 |

1. Blue tit – Pirio

|  | Wing length | | | | Tarsus length | | | | Mass | | | | Bill length | | | |
| --- | --- | --- | --- | --- | --- | --- | --- | --- | --- | --- | --- | --- | --- | --- | --- | --- |
|  | estimate | Lower CI | Upper CI | P | estimate | Lower CI | Upper CI | P | estimate | Lower CI | Upper CI | P | estimate | Lower CI | Upper CI | P |
| Intercept | 32.370 | 32.210 | 32.520 | <0.001 | 33.080 | 32.890 | 33.310 | <0.001 | 19.210 | 18.980 | 19.420 | <0.001 | 21.060 | 20.820 | 21.310 | <0.001 |
| Age | 0.554 | 0.455 | 0.655 | <0.001 | -0.042 | -0.122 | 0.043 | 0.348 | 0.222 | 0.074 | 0.367 | 0.002 | -0.058 | -0.244 | 0.100 | 0.490 |
| Age² | -0.088 | -0.116 | -0.060 | <0.001 | 0.004 | -0.017 | 0.024 | 0.750 | -0.035 | -0.075 | 0.006 | 0.088 | 0.010 | -0.037 | 0.053 | 0.686 |
| Sex | -1.385 | -1.464 | -1.317 | <0.001 | -0.879 | -0.998 | -0.761 | <0.001 | -0.295 | -0.406 | -0.172 | <0.001 | 0.439 | 0.313 | 0.552 | <0.001 |
| Age:Sex | -0.191 | -0.253 | -0.127 | <0.001 | 0.040 | -0.007 | 0.093 | 0.124 | -0.044 | -0.124 | 0.059 | 0.346 | 0.019 | -0.004 | 0.041 | 0.104 |
| Age²:Sex | 0.023 | 0.004 | 0.043 | 0.022 | -0.006 | -0.022 | 0.007 | 0.446 | 0.011 | -0.017 | 0.038 | 0.432 | 0.000 | -0.001 | 0.002 | 0.688 |
| Day |  |  |  |  |  |  |  |  | -0.008 | -0.026 | 0.009 | 0.324 | 0.000 | 0.000 | 0.000 | 0.252 |
| Day² |  |  |  |  |  |  |  |  | 0.001 | -0.001 | 0.002 | 0.390 | 0.054 | -0.055 | 0.168 | 0.308 |
| Day^3^ |  |  |  |  |  |  |  |  | 0.000 | 0.000 | 0.000 | 0.330 | -0.007 | -0.039 | 0.027 | 0.706 |
| Day:Sex |  |  |  |  |  |  |  |  | -0.003 | -0.013 | 0.009 | 0.582 | -0.001 | -0.015 | 0.013 | 0.886 |
| Day²:Sex |  |  |  |  |  |  |  |  | 0.001 | 0.000 | 0.001 | 0.276 | -0.001 | -0.002 | 0.000 | 0.112 |
| Day^3^:Sex |  |  |  |  |  |  |  |  | 0.000 | 0.000 | 0.000 | 0.166 | 0.000 | 0.000 | 0.000 | 0.134 |

1. Blue tit – Rouvière

|  | Wing length | | | | Tarsus length | | | | Mass | | | | Bill length | | | |
| --- | --- | --- | --- | --- | --- | --- | --- | --- | --- | --- | --- | --- | --- | --- | --- | --- |
|  | estimate | Lower CI | Upper CI | P | estimate | Lower CI | Upper CI | P | estimate | Lower CI | Upper CI | P | estimate | Lower CI | Upper CI | P |
| Intercept | 30.990 | 30.790 | 31.200 | <0.001 | 33.610 | 33.390 | 33.830 | <0.001 | 17.290 | 17.020 | 17.550 | <0.001 | 20.640 | 20.360 | 20.940 | <0.001 |
| Age | 0.633 | 0.539 | 0.745 | <0.001 | 0.024 | -0.047 | 0.111 | 0.554 | -0.001 | -0.116 | 0.145 | 0.940 | -0.025 | -0.181 | 0.168 | 0.772 |
| Age² | -0.174 | -0.221 | -0.129 | <0.001 | 0.001 | -0.030 | 0.035 | 0.986 | -0.031 | -0.097 | 0.021 | 0.304 | -0.026 | -0.103 | 0.050 | 0.480 |
| Sex | -1.201 | -1.291 | -1.116 | <0.001 | -1.015 | -1.119 | -0.912 | <0.001 | -0.145 | -0.277 | -0.042 | 0.014 | 0.413 | 0.283 | 0.540 | <0.001 |
| Age:Sex | -0.088 | -0.153 | -0.021 | 0.004 | -0.008 | -0.062 | 0.037 | 0.718 | 0.080 | -0.007 | 0.158 | 0.078 | 0.023 | -0.084 | 0.130 | 0.660 |
| Age²:Sex | 0.017 | -0.012 | 0.049 | 0.260 | -0.008 | -0.031 | 0.012 | 0.436 | -0.003 | -0.041 | 0.034 | 0.894 | 0.006 | -0.046 | 0.060 | 0.850 |
| Day |  |  |  |  |  |  |  |  | 0.015 | 0.002 | 0.028 | 0.032 | 0.027 | 0.012 | 0.045 | 0.004 |
| Day² |  |  |  |  |  |  |  |  | -0.001 | -0.001 | -0.001 | <0.001 | 0.000 | 0.000 | 0.001 | 0.188 |
| Day^3^ |  |  |  |  |  |  |  |  | 0.000 | 0.000 | 0.000 | 0.778 | 0.000 | 0.000 | 0.000 | 0.136 |
| Day:Sex |  |  |  |  |  |  |  |  | -0.027 | -0.035 | -0.018 | <0.001 | -0.007 | -0.018 | 0.003 | 0.164 |
| Day²:Sex |  |  |  |  |  |  |  |  | 0.001 | 0.001 | 0.001 | <0.001 | 0.000 | -0.001 | 0.000 | 0.030 |
| Day^3^:Sex |  |  |  |  |  |  |  |  | 0.000 | 0.000 | 0.000 | 0.842 | 0.000 | 0.000 | 0.000 | 0.086 |

1. Collared flycatcher

|  | Wing length | | | | Tarsus length | | | | Mass | | | | Bill length | | | |
| --- | --- | --- | --- | --- | --- | --- | --- | --- | --- | --- | --- | --- | --- | --- | --- | --- |
|  | estimate | Lower CI | Upper CI | P | estimate | Lower CI | Upper CI | P | estimate | Lower CI | Upper CI | P | estimate | Lower CI | Upper CI | P |
| (Intercept) | 37.820 | 37.690 | 37.950 | <0.001 | 32.890 | 32.790 | 33.000 | <0.001 | 9.845 | 9.695 | 10.000 | <0.001 |  |  |  |  |
| Age | 0.381 | 0.362 | 0.406 | <0.001 | 0.043 | 0.026 | 0.059 | <0.001 | 0.057 | 0.037 | 0.077 | <0.001 |  |  |  |  |
| Age² | -0.094 | -0.103 | -0.086 | <0.001 | -0.009 | -0.015 | -0.004 | <0.001 | -0.006 | -0.015 | 0.001 | 0.154 |  |  |  |  |
| Sex | -0.759 | -0.804 | -0.717 | <0.001 | 0.127 | 0.077 | 0.170 | <0.001 | 0.617 | 0.579 | 0.659 | <0.001 |  |  |  |  |
| Age:Sex | -0.096 | -0.126 | -0.066 | <0.001 | -0.019 | -0.041 | 0.003 | 0.108 | -0.063 | -0.088 | -0.034 | <0.001 |  |  |  |  |
| Age²:Sex | 0.024 | 0.012 | 0.036 | <0.001 | 0.008 | 0.000 | 0.017 | 0.066 | 0.011 | 0.000 | 0.022 | 0.050 |  |  |  |  |
| Day |  |  |  |  |  |  |  |  | -0.035 | -0.039 | -0.031 | <0.001 |  |  |  |  |
| Day² |  |  |  |  |  |  |  |  | 0.000 | 0.000 | 0.000 | 0.018 |  |  |  |  |
| Day:Sex |  |  |  |  |  |  |  |  | 0.000 | 0.000 | 0.000 | <0.001 |  |  |  |  |
| Day²:Sex |  |  |  |  |  |  |  |  | -0.071 | -0.076 | -0.066 | <0.001 |  |  |  |  |

1. Savannah sparrow

|  | Wing length | | | | Tarsus length | | | | Mass | | | | Bill length | | | |
| --- | --- | --- | --- | --- | --- | --- | --- | --- | --- | --- | --- | --- | --- | --- | --- | --- |
|  | estimate | Lower CI | Upper CI | P | estimate | Lower CI | Upper CI | P | estimate | Lower CI | Upper CI | P | estimate | Lower CI | Upper CI | P |
| Intercept | 27.180 | 27.020 | 27.360 | <0.001 | 31.130 | 30.810 | 31.370 | <0.001 | 13.310 | 13.060 | 13.540 | <0.001 | 22.680 | 22.410 | 22.960 | <0.001 |
| Age | 0.601 | 0.484 | 0.710 | <0.001 | 0.007 | -0.122 | 0.133 | 0.908 | 0.392 | 0.233 | 0.600 | <0.001 | 0.245 | 0.014 | 0.443 | 0.02 |
| Age² | -0.095 | -0.126 | -0.062 | <0.001 | 0.000 | -0.033 | 0.034 | 0.954 | -0.074 | -0.122 | -0.015 | 0.006 | -0.017 | -0.080 | 0.032 | 0.574 |
| Sex | -1.582 | -1.642 | -1.513 | <0.001 | -0.781 | -0.871 | -0.680 | <0.001 | -0.451 | -0.561 | -0.332 | <0.001 | -0.688 | -0.823 | -0.555 | <0.001 |
| Age:Sex | -0.148 | -0.224 | -0.075 | <0.001 | 0.023 | -0.063 | 0.098 | 0.588 | -0.210 | -0.316 | -0.079 | <0.001 | -0.128 | -0.257 | 0.007 | 0.064 |
| Age²:Sex | 0.015 | -0.008 | 0.037 | 0.186 | 0.001 | -0.020 | 0.028 | 0.938 | 0.047 | 0.010 | 0.085 | 0.018 | 0.005 | -0.038 | 0.044 | 0.826 |
| Day |  |  |  |  |  |  |  |  | -0.016 | -0.023 | -0.010 | <0.001 | 0.005 | -0.004 | 0.013 | 0.226 |
| Day² |  |  |  |  |  |  |  |  | 0.000 | 0.000 | 0.001 | 0.002 | -0.001 | -0.001 | 0.000 | <0.001 |
| Day^3^ |  |  |  |  |  |  |  |  | 0.000 | 0.000 | 0.000 | 0.1 | 0.000 | 0.000 | 0.000 | 0.006 |
| Day:Sex |  |  |  |  |  |  |  |  | 0.004 | 0.001 | 0.009 | 0.026 | 0.000 | -0.005 | 0.005 | 0.912 |
| Day²:Sex |  |  |  |  |  |  |  |  | -0.001 | -0.001 | -0.001 | <0.001 | 0.000 | 0.000 | 0.000 | 0.002 |
| Day^3^:Sex |  |  |  |  |  |  |  |  | 0.000 | 0.000 | 0.000 | <0.001 | 0.000 | 0.000 | 0.000 | 0.068 |

1. House sparrow

|  | Wing length | | | | Tarsus length | | | | Mass | | | | Bill length | | | |
| --- | --- | --- | --- | --- | --- | --- | --- | --- | --- | --- | --- | --- | --- | --- | --- | --- |
|  | estimate | Lower CI | Upper CI | P | estimate | Lower CI | Upper CI | P | estimate | Lower CI | Upper CI | P | estimate | Lower CI | Upper CI | P |
| (Intercept) | 35.140 | 34.910 | 35.380 | <0.001 | 22.160 | 21.910 | 22.430 | <0.001 | 14.820 | 14.530 | 15.110 | <0.001 | 22.650 | 22.050 | 23.230 | <0.001 |
| Age | 0.120 | 0.069 | 0.175 | <0.001 | 0.071 | 0.015 | 0.118 | 0.008 | 0.079 | 0.000 | 0.167 | 0.060 | 0.091 | 0.002 | 0.188 | 0.050 |
| Age² | -0.036 | -0.058 | -0.011 | 0.002 | 0.012 | -0.009 | 0.033 | 0.302 | -0.018 | -0.054 | 0.023 | 0.342 | -0.048 | -0.085 | -0.014 | 0.014 |
| Sex | 1.226 | 1.075 | 1.363 | <0.001 | 0.205 | 0.024 | 0.435 | 0.052 | 0.200 | 0.004 | 0.413 | 0.060 | -0.105 | -0.326 | 0.083 | 0.308 |
| Age:Sex | 0.094 | 0.036 | 0.170 | 0.010 | -0.011 | -0.070 | 0.049 | 0.730 | -0.071 | -0.184 | 0.029 | 0.210 | 0.062 | -0.055 | 0.179 | 0.280 |
| Age²:Sex | 0.004 | -0.028 | 0.034 | 0.836 | -0.018 | -0.046 | 0.008 | 0.228 | 0.025 | -0.024 | 0.076 | 0.316 | 0.030 | -0.015 | 0.082 | 0.234 |
