# Supplementary material for: Assessing Multivariate Constraints to Evolution across Ten Long-Term Avian Studies
Source: PLoS One. 2014 Mar 7;9(3):e90444. doi: 10.1371/journal.pone.0090444 (PMC3946496; doi:10.1371/journal.pone.0090444)
Supplement: Table S2 — Estimates of heritabilities (with traits standardized to the variance) for each population. (DOC) [file pone.0090444.s004.doc]

**Table S2**: Estimates of heritabilities (with traits standardized to the variance) for each population with their 95% confidence interval.

|  | Red billed gull | | | Great reed warbler | | | Barn swallow - Badajoz | | |
| --- | --- | --- | --- | --- | --- | --- | --- | --- | --- |
|  | posterior mode | Lower 95%CI | Upper 95%CI | posterior mode | Lower 95%CI | Upper 95%CI | posterior mode | Lower 95%CI | Upper 95%CI |
| Wing | 0.546 | 0.457 | 0.615 | 0.502 | 0.363 | 0.594 | 0.331 | 0.173 | 0.438 |
| Tarsus | 0.404 | 0.289 | 0.474 | 0.552 | 0.375 | 0.667 | 0.13 | 0.062 | 0.177 |
| Mass | 0.188 | 0.149 | 0.234 | 0.215 | 0.118 | 0.293 | 0.188 | 0.102 | 0.254 |
| Bill | 0.419 | 0.374 | 0.472 | 0.594 | 0.421 | 0.718 | 0.115 | 0.063 | 0.18 |

|  | Barn swallow - Kraghede | | | Blue tit - Muro | | | Blue tit - Pirio | | |
| --- | --- | --- | --- | --- | --- | --- | --- | --- | --- |
|  | posterior mode | Lower 95%CI | Upper 95%CI | posterior mode | Lower 95%CI | Upper 95%CI | posterior mode | Lower 95%CI | Upper 95%CI |
| Wing | 0.505 | 0.333 | 0.678 | 0.247 | 0.174 | 0.367 | 0.216 | 0.152 | 0.296 |
| Tarsus | 0.22 | 0.117 | 0.35 | 0.418 | 0.277 | 0.555 | 0.513 | 0.338 | 0.644 |
| Mass | 0.284 | 0.143 | 0.443 | 0.299 | 0.167 | 0.418 | 0.281 | 0.164 | 0.365 |
| Bill | 0.127 | 0.05 | 0.203 | 0.285 | 0.164 | 0.406 | 0.266 | 0.159 | 0.407 |

|  | Blue tit - Rouvière | | | Collared flycatcher | | | Savannah sparrow | | |
| --- | --- | --- | --- | --- | --- | --- | --- | --- | --- |
|  | posterior mode | Lower 95%CI | Upper 95%CI | posterior mode | Lower 95%CI | Upper 95%CI | posterior mode | Lower 95%CI | Upper 95%CI |
| Wing | 0.319 | 0.24 | 0.38 | 0.312 | 0.267 | 0.363 | 0.29 | 0.203 | 0.39 |
| Tarsus | 0.597 | 0.486 | 0.675 | 0.438 | 0.393 | 0.501 | 0.276 | 0.21 | 0.438 |
| Mass | 0.36 | 0.264 | 0.425 | 0.189 | 0.157 | 0.238 | 0.244 | 0.148 | 0.329 |
| Bill | 0.387 | 0.274 | 0.517 | 0.051 | 0.026 | 0.083 | 0.454 | 0.321 | 0.587 |

|  | House sparrow | | |
| --- | --- | --- | --- |
|  | posterior mode | Lower 95%CI | Upper 95%CI |
| Wing | 0.479 | 0.313 | 0.571 |
| Tarsus | 0.364 | 0.256 | 0.558 |
| Mass | 0.346 | 0.223 | 0.445 |
| Bill | 0.351 | 0.17 | 0.515 |
