# Supplementary material for: Assessing Multivariate Constraints to Evolution across Ten Long-Term Avian Studies
Source: PLoS One. 2014 Mar 7;9(3):e90444. doi: 10.1371/journal.pone.0090444 (PMC3946496; doi:10.1371/journal.pone.0090444)
Supplement: Table S3 — Predicted responses to selection (×100) in multivariate and univariate frameworks, and the angle between selection and predicted response to selection (Angle(R, β)). (DOC) [file pone.0090444.s005.doc]

**Table S3**: Predicted responses to selection (×100) in multivariate and univariate frameworks, and the angle between selection and predicted response to selection (Angle(R, β)). Estimates are given with the 95% confidence interval. Bold: predictions that are significantly different from zero.

|  |  | Multivariate prediction | | | Univariate prediction | | | Angle(R, β) | | |
| --- | --- | --- | --- | --- | --- | --- | --- | --- | --- | --- |
| Population | Trait | estimate | Low CI | High CI | estimate | Low CI | High CI | estimate | Low CI | High CI |
| Red billed gull | Wing | 0.0542 | -0.0730 | 0.1784 | 0.0338 | -0.0820 | 0.1831 | 36.06 | 9.09 | 45.63 |
|  | Tarsus | 0.1181 | -0.0339 | 0.2263 | 0.0889 | -0.0433 | 0.2310 |  |  |  |
|  | Mass | 0.0072 | -0.1453 | 0.1776 | -0.1448 | -0.2961 | 0.0241 |  |  |  |
|  | Bill | 0.0958 | -0.0359 | 0.2159 | 0.0718 | -0.0624 | 0.2041 |  |  |  |
| Great reed warbler | Wing | 0.0108 | -0.0917 | 0.0803 | -0.0018 | -0.0780 | 0.0801 | 36.50 | 10.09 | 50.70 |
|  | Tarsus | 0.0972 | -0.0092 | 0.2358 | **0.1677** | **0.0402** | **0.2911** |  |  |  |
|  | Mass | -0.0385 | -0.1818 | 0.1192 | **-0.1340** | **-0.2956** | **-0.0358** |  |  |  |
|  | Bill | 0.0386 | -0.1073 | 0.2284 | 0.0470 | -0.1431 | 0.1630 |  |  |  |
| Barn swallow - Badajoz | Wing | 0.0123 | -0.0136 | 0.0330 | 0.0067 | -0.0171 | 0.0293 | 48.34 | 21.25 | 61.71 |
|  | Tarsus | 0.0042 | -0.0160 | 0.0259 | 0.0042 | -0.0167 | 0.0209 |  |  |  |
|  | Mass | 0.0324 | -0.0313 | 0.0831 | 0.0108 | -0.0404 | 0.0667 |  |  |  |
|  | Bill | 0.0066 | -0.0169 | 0.0567 | 0.0090 | -0.0214 | 0.0454 |  |  |  |
| Barn swallow - Kraghede | Wing | 0.0056 | -0.0418 | 0.0555 | -0.0119 | -0.0557 | 0.0418 | 45.27 | 11.97 | 60.98 |
|  | Tarsus | 0.0564 | -0.0076 | 0.1443 | **0.0631** | **0.0026** | **0.1282** |  |  |  |
|  | Mass | -0.0118 | -0.0886 | 0.1258 | -0.0229 | -0.1014 | 0.0813 |  |  |  |
|  | Bill | 0.0046 | -0.0286 | 0.0656 | 0.0126 | -0.0193 | 0.0539 |  |  |  |
| Blue tit - Muro | Wing | 0.0056 | -0.0109 | 0.0222 | 0.0043 | -0.0098 | 0.0190 | 36.50 | 9.26 | 48.78 |
|  | Tarsus | 0.0173 | -0.0044 | 0.0487 | 0.0155 | -0.0100 | 0.0444 |  |  |  |
|  | Mass | 0.0075 | -0.0302 | 0.0373 | -0.0238 | -0.0486 | 0.0131 |  |  |  |
|  | Bill | **0.0240** | **0.0033** | **0.0521** | **0.0229** | **0.0037** | **0.0444** |  |  |  |
| Blue tit - Pirio | Wing | 0.0013 | -0.0131 | 0.0184 | 0.0087 | -0.0059 | 0.0210 | 37.08 | 9.96 | 54.06 |
|  | Tarsus | 0.0060 | -0.0269 | 0.0438 | 0.0196 | -0.0146 | 0.0585 |  |  |  |
|  | Mass | -0.0374 | -0.0796 | 0.0039 | **-0.0499** | **-0.1079** | **-0.0234** |  |  |  |
|  | Bill | 0.0027 | -0.0149 | 0.0259 | 0.0102 | -0.0059 | 0.0249 |  |  |  |
| Blue tit - Rouvière | Wing | 0.0073 | -0.0072 | 0.0227 | 0.0048 | -0.0075 | 0.0205 | 24.74 | 6.60 | 43.27 |
|  | Tarsus | **0.0322** | **0.0101** | **0.0668** | **0.0305** | **0.0114** | **0.0713** |  |  |  |
|  | Mass | 0.0084 | -0.0340 | 0.0441 | -0.0282 | -0.0671 | 0.0129 |  |  |  |
|  | Bill | 0.0068 | -0.0127 | 0.0316 | 0.0052 | -0.0143 | 0.0263 |  |  |  |
| Collared flycatcher | Wing | 0.0088 | -0.0016 | 0.0200 | **0.0145** | **0.0017** | **0.0238** | 39.90 | 15.77 | 50.82 |
|  | Tarsus | 0.0092 | -0.0076 | 0.0281 | 0.0209 | -0.0005 | 0.0360 |  |  |  |
|  | Mass | **-0.0211** | **-0.0511** | **-0.0022** | **-0.0490** | **-0.0687** | **-0.0234** |  |  |  |
|  | Bill | -0.0043 | -0.0201 | 0.0132 | -0.0062 | -0.0227 | 0.0079 |  |  |  |
| Savannah sparrow | Wing | 0.0118 | -0.0321 | 0.0506 | 0.0121 | -0.0231 | 0.0515 | 22.10 | 10.57 | 44.46 |
|  | Tarsus | -0.0373 | -0.0865 | 0.0218 | -0.0367 | -0.0923 | 0.0131 |  |  |  |
|  | Mass | -0.0344 | -0.1392 | 0.0496 | **-0.0643** | **-0.1705** | **-0.0080** |  |  |  |
|  | Bill | **0.1007** | **0.0091** | **0.1770** | **0.1418** | **0.0545** | **0.2140** |  |  |  |
| House sparrow | Wing | 0.0158 | -0.0471 | 0.0735 | -0.0110 | -0.0723 | 0.0596 | 47.04 | 20.01 | 64.47 |
|  | Tarsus | 0.0572 | -0.0589 | 0.1724 | 0.1129 | -0.0177 | 0.2559 |  |  |  |
|  | Mass | 0.0257 | -0.1233 | 0.1822 | -0.0480 | -0.2002 | 0.1073 |  |  |  |
|  | Bill | -0.0296 | -0.1644 | 0.0707 | -0.0449 | -0.1733 | 0.0496 |  |  |  |
